# Can air pollution affect tear film stability? a cross-sectional study in the aftermath of an explosion accident

**DOI:** 10.1186/1471-2458-11-235

**Published:** 2011-04-14

**Authors:** Bente E Moen, D Norbäck, G Wieslander, JV Bakke, N Magerøy, JT Granslo, Å Irgens, M Bråtveit, BE Hollund, T Aasen

**Affiliations:** 1 Occupational and Environmental Medicine, Department of Public Health and Primary Health Care, University of Bergen, Kalfarveien 31. NO-5020 Bergen, Norway; 2 Occupational Medicine, Haukeland University Hospital, Bergen, Norway; 3 Department of Medical Sciences, Occupational and Environmental Medicine, Uppsala University and University Hospital, SE-751 85 Uppsala, Sweden; 4 The Norwegian Labour Inspection Authority, Gjøvik, Norway; 5 Department of Energy and Process Engineering, Norwegian University of Science and Technology, NO-7034 Trondheim, Norway

## Abstract

**Background:**

After an explosion and fire in two tanks containing contaminated oil and sulphur products in a Norwegian industrial harbour in 2007, the surrounding area was polluted. This caused an intense smell, lasting until the waste was removed two years later. The present study reports examinations of tear film break up time among the population. The examinations were carried out because many of the people in the area complained of sore eyes. The purpose of the study was to assess the relationship between living or working close to the polluted area and tear film stability one and a half years after the explosion.

**Methods:**

All persons working or living in an area less than six kilometres from the explosion site were invited to take part in the study together with a similar number of persons matched for age and gender living more than 20 kilometres away. Three groups were established: workers in the explosion area and inhabitants near the explosion area (but not working there) were considered to have been exposed, and inhabitants far away (who did not work in the explosion area) were considered to be unexposed. A total of 734 people were examined, and the response rate was 76 percent. Tear film stability was studied by assessing non-invasive break-up time (NIBUT) using ocular microscopy. In addition Self-reported Break Up Time (SBUT) was assessed by recording the time the subject could keep his or hers eyes open without blinking when watching a fixed point on a wall. Background information was obtained using a questionnaire. Non-parametric Wilcoxon-Mann-Whitney-tests with exact p-values and multiple logistic regression analyses were performed.

**Results:**

Both NIBUT and SBUT were shorter among the male exposed workers than among the inhabitants both near and far away from the explosion area. This was also found for SBUT among males in a multiple logistic regression analysis, adjusting for age and smoking.

**Conclusions:**

Reduced tear film stability was found among workers in an area where an explosion accident had occurred.

## Background

On the 24th May 2007, a tank containing contaminated oil exploded and caught fire together with a neighbouring tank in an industrial harbour area on the west coast of Norway. Black smoke spread over an area several hundred metres north of the harbour area, as the wind was blowing in that direction. The fire was completely extinguished the next day. Combustion products from the fire as well as coker gasoline mixed with sulphur products from the tanks caused contamination and an intense smell. The smell affected employees in nearly workplaces nearby and people who lived in houses several kilometres away. It took more than two years before the area was cleaned and the contaminated soil was removed. The unpleasant smell was present in the area during this whole period, and it affected both the population living close by and those who lived four to six kilometres away. Twelve measurements taken in ambient air a few metres away from the exploded tanks six weeks after the explosion, showed mercaptanes at a level of about 1-2 percent of the Norwegian limit value of 1 mg/m^3 ^for workplaces [1]. Hydrocarbon compounds were also measured in the air close to the explosion site, showing similar low levels of toluene and xylenes. These compounds were also measured further away from the explosion site, showing zero levels in the air some hundred metres away. Very few samples were taken in total. The tanks belonged to a company engaged in the disposal of discarded oil products. Another company of the same type was located in the harbour areas, as well as a quarry, cement company and various transport companies.

Two local general practitioners in the area carried out interview surveys among the population. Of the 144 interviewed adults who lived close to the explosion area in 2007, 22 per cent reported sore and irritated eyes [2]. A re-examination of 128 of these individuals in 2008 revealed similar problems among 15 percent of them [2]. Other symptoms were present as well, such as headaches, sore throats and nausea. However, it is unclear what methods were used in these surveys are not clear, for example how the questions were worded, and the results are generally difficult to interpret.

Previous studies have reported that individuals in areas exposed to pollutants may report ocular symptoms [3,4] both caused by outdoor exposure, indoor exposure as a result of cooking [5] or office work [6,7]. Eye symptoms have also been reported among participants in a clean-up operation after an oil spill [8,9]. These studies have not examined long term effects of pollution on the eyes.

The tank explosion gave rise to extensive coverage in the press and local broadcast media as long as the smell was present in the area. The presence of symptoms such as headaches, nausea, sore eyes and throats was often mentioned as a problem among the local population. The Norwegian health authorities implemented a health examination of the inhabitants in the area, in order to establish the size of the problem and to provide help where this was needed. To avoid information bias, objective measures of health problems were required, since the reports in the media could influence the answers in a questionnaire study. Tear film stability has been used as an objective measure for air borne pollutants, both indoors [10], outdoors [11] and in experimental studies [12,13]. The studied population was not familiar with this type of test. The purpose of the present study was to assess the relationship between living or working close to an area contaminated by coker gasoline, sulphur products and combustion products and tear film stability one and a half years after the explosion. Our hypothesis was that persons who had been working or living close to the polluted area would have a shorter tear film break-up-time than persons who lived farther away.

## Methods

### Design, study area and population

In 2008, about one and a half years after the explosion, everyone working or living in an area less than six kilometres from the explosion site at the time of the explosion was asked to participate in this cross-sectional study. The distance of six kilometres was a natural choice, as this area contained a group of houses. There were no houses in the area immediately beyond six kilometres from the harbour. A similar number of persons matched for age and gender, living more than 20 kilometres away were asked to participate as an unexposed group. The area in which both the exposed and unexposed participants in this study lived was far away from any city and had very little road traffic.

The names, age and addresses of the persons chosen for the study were found in the National Population Registry of Norway. Persons who had worked close to the explosion were found by contacting the different work places in the harbour area. Three groups were established: 1) Workers in the explosion area, 2) inhabitants near the explosion area (but not working in the explosion area) and 3) inhabitants more than 20 kilometres away (and not working in the explosion area). The workers and inhabitants near the explosion area were considered to have been exposed to pollution, while the third group was considered to be unexposed. In total, 1016 persons were asked to participate by personal letter sent by mail.

### Examinations

The respondents completed a questionnaire which they brought along with them to a health examination. Of the participants, 485 were examined in the local area, while for practical reasons, 34 were examined in the nearest city, Bergen. This publication only reports the eye examinations and relevant background information from the questionnaire. Several other health parameters were examined and will be reported elsewhere. The information from the questionnaire used in the present study was allergy in the family, having ever experienced allergy, the presence of carpets, pets and moisture at home, present smoking, participation in fire fighting after the explosion, participation in cleaning up the area after the explosion, occupation and workplace. The occupations were classified into five groups: Office work, industrial work, service work, agriculture and transport. Tear film stability was studied by assessing non-invasive break-up time (NIBUT) using ocular microscopy (Keeler TearScope^®^, Keeler Instruments, Clewer Hill Road, Windsor, Berkshire SL4 4AA, UK), based on a grid of equidistant circles of light that are blurred by tear film break up [14]. In addition Self-reported Break Up Time (SBUT) was assessed by recording the time the subject could keep his or her eyes open without blinking, when watching a fixed point on a wall. This method has been used previously [10]. It has been shown to correlate well with the fluorescein method for break-up time (BUT) [15]. The recording of NIBUT and SBUT was stopped at 60 seconds. The average for three tests was registered for both NIBUT and SBUT. Contact lens wearers (30 persons) were not assessed. The participants were examined using a standardised procedure after they had been indoors for minimum half an hour. Five physicians participated in the examinations. The Regional Committee for Medical Research Ethics of Western Norway and the Norwegian Social Science Data Services approved the study.

### Statistical analysis

As the tear film data were not normally distributed, the non-parametric Wilcoxon-Mann-Whitney test with exact p-value was used for comparisons of groups. Multiple logistic regression analyses were performed separately with NIBUT and SBUT as dependent variables. In these analyses NIBUT and SBUT were categorized with cut-off points of 20 seconds and 30 seconds, respectively, based on normative values from previous studies [10,16]. Exposure group (non-exposed - living far away - as reference), age, years of education and present smoking were included in the regression model. SPSS version 15.0 was used for the analyses and the significance level was set below 0.05.

## Results

The response rate was 76 percent in the exposed group and 59 in the unexposed group. A total of 734 persons were examined. The study group in the present study was restricted to the 18-67 age group, as this is the age range of the Norwegian working population; 519 persons, 38% women and 62% men. Fifty-three of the workers in the explosion area had participated in fire fighting and 42 in the cleaning operations after the explosion. They had only been present at the explosion site for one or two days. Only one of these workers was female.

The male exposed worker group was slightly older and contained more smokers than the unexposed group (Table 1). There were no differences between the groups as regards allergy in the family, ever having experienced allergy, the presence of carpets, pets and moisture at home or present medication. All five categories of occupations were similarly represented in all three exposure groups. However, the majority of male exposed workers were production workers, mechanics and drivers, while the majority of female exposed workers were office and service workers.

**Table 1:** Number of participants, age and smoking habits among men and women (18-67 years) in three exposure groups related to an explosion causing environmental pollution

	Exposure group*	Men	Women
**Number of participants**			
	UI	83	66
	EI	67	77
	EW	184	42
	Total	334	185
**Age-mean (SD)**			
	UI	42 (12)	46 (13)
	EI	48 (17)	44 (14)
	EW	44 (12)	43 (11)
	Total	44 (13)	43 (13)
**Present smokers (%)**			
	UI	28	18
	EI	31	34
	EW	36	34
	Total	32	25

*UI = Unexposed inhabitants far from the explosion area

*EI = Exposed inhabitants near the explosion area

*EW = Exposed workers at the explosion area

Both NIBUT and SBUT were shorter among the exposed workers than among the inhabitants near and far from the explosion area (Table 2). The difference was significant for men (Wilcoxon-Mann-Whitney-test with exact p-value = 0.001), but not for women. The women had shorter SBUT than men (Wilcoxon-Mann-Whitney-test, with exact p-value = 0.01), but there was no difference between the genders for NIBUT.

**Table 2:** Non-invasive break-up time (NIBUT) and self-reported break-up time (SBUT) measured in seconds among men and women (18-67 years) in three exposure groups related to an explosion causing environmental pollution

	Exposure group*	Men Mean (SD)		Median (range)		Women Mean (SD)		Median (range)	
**NIBUT**									
	UI	43	(19)	32	(7-60)	42	(21)	52	(7-60)
	EI	44	(18)	48	(9-60)	37	(20)	32	(6-60)
	EW	36	(19)	30	(5-60)†	36	(19)	37	(4-60)
	Total	40	(19)	37	(5-60)	39	(20)	39	(5-60)
**SBUT**									
	UI	44	(21)	26	(4-60)	33	(22)	25	(4-60)
	EI	42	(21)	53	(4-60)	29	(21)	20	(6-60)
	EW	35	(22)	28	(4-60)†	31	(22)	21	(2-60)
	Total	38	(22)	39	(4-60)	31	(22)	21	(2-60)

*UI = Unexposed inhabitants far from the explosion area

*EI = Exposed inhabitants near the explosion area

*EW = Exposed workers at the explosion area

† Significant differences between workers and inhabitants near pollution and also between workers and inhabitants far from pollution, Wilcoxon-Mann-Whitney-test, with exact p-values; p = 0.001 for both comparisons.

Low SBUT was related to high exposure (workers) among the male participants, OR = 2.0, 95% CI 1.1-3.8, shown by multiple logistic regression analysis with SBUT as the dependent variable, including exposure group, age, years of education and present smoking in the model. In a similar analysis of NIBUT, the findings were not significant: OR = 1.6, 95% CI 0.7 - 3.5.

For women, no significant relationships were found in a similar analysis with SBUT or NIBUT as the dependent variable including exposure group (non-exposed - living far away - as reference), age, years of education and present smoking in the model: OR = 0.9, 95% CI 0.4-1.9 for SBUT and OR = 0.9, 95% CI 0.4-2-1 for NIBUT.

Analysing only the male working population (excluding 13% who were not in employment), and also including the five occupational categories as five separate variables (0 or 1) in the model, did not influence the estimates. Similarly, including participation in fire fighting, cleaning up the area or place of examination did not influence the results.

## Discussion

Male exposed workers in this study had shorter tear film break-up time than the other male groups in the study. As this was a cross-sectional study, and no examination of the population was carried out before the explosion, it is difficult to know whether or not this finding is caused by pollution resulting from the explosion. However, the contamination from the explosion consisted of different hydrocarbon compounds mixed with sulphur products, and the smell and pollution in the area lasted for two years. Malodorous sulphur air pollutants have previously been described as being related to irritation of the eyes [17] and a relationship here is possible. Other studies have shown possible effects of outdoor air pollution on the tear film [11,18]. In a study of air pollution from Delhi [11], tear film abnormalities were suggested to be related to outdoor air pollution over a longer period, not only as a result of acute exposure. Similarly, an effect of long-term outdoor air pollution might be present in our present study. However, we cannot rule out that the shorter tear film break-up time among the exposed workers is related to other kinds of pollution than that caused by the explosion pollution. Although our three exposure groups were established in relation to the site of the explosion, the differences in tear film might be related to other pollution sources than the explosion, especially since the time from the explosion happened until the examinations were performed was quite long. Moreover, the group described here as the 'highest exposed' were workers who might be exposed to dust and chemicals during their ordinary work.

The tear film break-up time registered in this study was generally rather long compared with some previous studies [16,19], but it was in line with others [10,14,20]. Differences in these figures might be due to differences in methodology.

The female participants had shorter break-up time values than the males. This has been registered in previous studies as well [7], although not in all of them [20]. We lack the detailed information required to explain this finding. One possibility is that it might be related to differences in type of work. The male workers might be more likely to be outdoors during their work than the female workers. This could have caused different exposure levels to air pollutants. Moreover, the female working group was much smaller than the male group, and this could also have influenced the results. Previous studies have suggested type of work [7] or use of eye make-up [21] as possible factors causing gender difference in tear film stability.

The strengths of the study are a high response rate and an objective examination type. The response rate was lower among the unexposed inhabitants, introducing the possibility of selection bias. However, any possible effect from this was reduced as we were able to examine confounding factors such as allergy, exposure at home and smoking. Furthermore, while the groups were initially matched for age, we controlled for age in the regression analyses since the response rate differed between the groups. Most previous studies of tear film examinations suggest a clear relationship between eye irritation symptoms and objective alterations of the tear film [22,23], making these examinations relevant to this study. However, some studies suggest a low degree of association between findings from similar examinations and eye symptoms [24,25]. On the other hand, these studies examined patients with dry eyes, not people with eye irritation due to pollution, and they used fluorescein tear break-up time. Moreover, the direct association between objective measurements and subjective symptoms was not the issue in the present study.

Although SBUT has some elements of subjective reporting, it is a more objective measure than, for instance, self-reported symptoms. NIBUT is also an objective method performed by physicians, but the interpretations of the results can differ more for NIBUT than for SBUT. This might be the explanation for the differences found in the SBUT and NIBUT results. Furthermore, the examinations were not performed blindly, introducing the possibility of bias.

The study would have been improved if the outdoor air had been systematically monitored, both at the time of the explosion and afterwards. Exposure data for the workers in the explosion area would also have been of great interest. This kind of monitoring in the areas from which the different groups of participants in the present study were taken would have made it easier to draw clear conclusions from the present study.

Despite the weaknesses of the study, we suggest tear film examinations as a method for examining effects of airborne pollution outdoors and in polluting industrial workplaces in future studies. SBUT might be a simpler examination to perform than NIBUT, but both types of examinations are relatively easy to perform. The measurements should be started shortly after the beginning of the exposure, and follow-up studies should be planned. The pollutants in the air should be monitored Studies of this kind could be important in relation to the implementation of preventive measures to reduce health problems associated with pollution.

## Conclusions

Reduced tear film stability was found among workers in an area where a polluting explosion accident had occurred.

## Competing interests

The authors declare that they have no competing interests.

## Authors' contributions

BEM, MB, TAA, JVB and BEH planned the study. BEH and NM carried out the field work, and NM and JTG performed the examinations. DN, GW, ÅI and BEM analysed the data. All authors participated in the discussion of the results. All authors read and approved the final version of the manuscript.

## Pre-publication history

The pre-publication history for this paper can be accessed here:

http://www.biomedcentral.com/1471-2458/11/235/prepub

## References

[B1] Rasmussen S Luftprøver for analyse av svovelholdige forbindelser og løsemidler - Vest Tank 2009 Rapport X-lab, Bergen, Norway (Report in Norwegian)

[B2] Tande RM Norman T Asheim TK Midtbø M Berg AL Rapport om helseplagar i Gulen og Masfjorden kommunar etter ulukka i Vest Tank sitt anlegg i Sløvåg 24.05.2007 2008 (Report in Norwegian)

[B3] Klopfer J Effects of environmental air pollution on the eye J Am Optom Assoc 1989 60 773 8 2685084

[B4] Saha A Kulkarni PK Shah A Patel M Saiyed HN Ocular morbidity and fuel use: an experience from India Occup Environ Med 2005 62 66 9 10.1136/oem.2004.015636 15613613 PMC1740843

[B5] Ellegard A Tears while cooking: An indicator of indoor air pollution and related health effects in developing countries Environ Res 1997 75 12 22 10.1006/enrs.1997.3771 9356190

[B6] Wieslander G Norbäck D Venge P Changes of symptoms, tear film stability and eosinophilic cationic protein in nasal lavage fluid after re-exposure to a damp office building with a history of flooding Indoor Air 2007 17 19 27 10.1111/j.1600-0668.2006.00441.x 17257149

[B7] Bakke JV Wieslander G Norbäck D Moen BE Atopy, symptoms and indoor environmental perceptions, tear film stability, nasal patency and lavage biomarkers in university staff Int Arch Occup Environ Health 2008 81 861 72 10.1007/s00420-007-0280-2 18066577

[B8] Suárez B Lope V Pérez-Gomez B Aragonés N Rodríguez-Artalejo F Marqués F Guzmán A Viloria LJ Carrasco JM Martín-Moreno JM López-Abente G Pollan M Acute health problems among subjects involved in the cleanup operation following the Prestige oil spill in Asturias and Cantabria (Spain) Environ Res 2005 99 413 424 10.1016/j.envres.2004.12.012 16307984

[B9] Janjua NZ Kasi PM Nawaz H Farooqui SZ Khuwaja UB Najam-ul-Hassan Jafri SN Lutfi SA Kadir MM Sathiakumar N Acute health effects of the Tasman Spirit oil spill on residents of Karachi, Pakistan BMC Public Health 2006 3 84 10.1186/1471-2458-6-84 PMC1484477 16584541

[B10] Wieslander G Norback D Nordstrom K Walinder R Venge P Nasal and ocular symptoms, tear film stability and biomarkers in nasal lavage, in relation to building-dampness and building design in hospitals Int Arch Occup Environ Health 1999 72 451 461 10.1007/s004200050398 10541910

[B11] Gupta SK Gupta V Joshi S Tandon R Subclinically dry eyes in urban Delhi: an impact of air pollution? Ophthalmologica 2002 216 368 71 10.1159/000066183 12424406

[B12] Zhao J Wollmer P Air pollutants and tear film stability - a method for experimental evaluation Clin Physiol 2000 21 282 286 10.1046/j.1365-2281.2001.00329.x 11380526

[B13] Novaes P Hilário do Nascimento Saldiv P Kara-José N Macchione M Matsuda M Racca L Berra A Ambient levels of air pollution induce goblet-cell hyperplasia in human conjunctival epithelium Environ Health Perspect 2007 115 1753 1756 10.1289/ehp.10363 18087595 PMC2137119

[B14] Mengher LS Bron AJ Tonge SR Gilbert DJ A non-invasive instrument for clinical assessment of the pre-corneal tear film stability Curr Eye Res 1985 4 1 7 10.3109/02713688508999960 3979089

[B15] Wyon NM Wyon DP Measurement of acute response to draught in the eye Acta Ophthalmol 1987 65 385 392 10.1111/j.1755-3768.1987.tb07011.x 3310505

[B16] Guillon M Styles E Guillon JP Maissa C Preocular tear film caracteristics of nonwearers and soft contact lens wearers Optom Vis Sci 1997 74 273 9 10.1097/00006324-199705000-00022 9219285

[B17] Haahtela T Marttila O Vilkka V Jäppinen P Jakkola JJK The South Karelia Air Pollution Study: acute health effects of malodorous sulfur air pollutants released by a pulp mill Am J Publ Health 1992 82 603 605 10.2105/AJPH.82.4.603 PMC1694105 1546787

[B18] Versura P Profazio V Cellini M Torreggiani A Caramazza R Eye discomfort and air pollution Ophthalmologica 1999 213 103 9 10.1159/000027401 9885386

[B19] Albietz JM Dry eye, an update on clinical diagnosis, management and promising new treatments Clin Exp Optom 2001 84 4 18 10.1111/j.1444-0938.2001.tb04930.x 12366339

[B20] Tonge SR Hunsaker J Holly FJ Non-invasive assessment of tear film break-up time in a group of normal subjects - implications for contact lens wear J Br Contact Lens Assoc 1991 14 201 205 10.1016/0141-7037(91)80022-E

[B21] Frank C Bach E Skov P Prevalence of objective eye manifestations in people working in office buildings with different prevalence of the sick building syndrome compared with the general population Int Arch Occup Environ Health 1993 65 65 69 10.1007/BF00586061 8354577

[B22] Wolkoff P Nøjgaard JK Franck C Skov P The modern office environment desiccate the eyes? Indoor Air 2006 16 258 265 10.1111/j.1600-0668.2006.00429.x 16842606

[B23] Bakke JV Wieslander G Norbäck D Moen BE Atopy, symptoms and indoor environmental perceptions, tear film stability, nasal patency and lavage biomarkers in university staff Int Arch Occup Environ Health 2008 81 861 72 10.1007/s00420-007-0280-2 18066577

[B24] Nichols KK Nichols JJ Mitchell GL The lack of association between signs and symptoms in patients with dry eye disease Cornea 2004 23 762 770 10.1097/01.ico.0000133997.07144.9e 15502475

[B25] Nichols KK Mitchell GL Zadnik K The repeatability of clinical measurements of dry eye Cornea 2004 23 272 285 10.1097/00003226-200404000-00010 15084861

